# Development and Processing of Continuous Flax and Carbon Fiber-Reinforced Thermoplastic Composites by a Modified Material Extrusion Process

**DOI:** 10.3390/ma14092332

**Published:** 2021-04-30

**Authors:** Sebastian Kuschmitz, Arne Schirp, Johannes Busse, Hagen Watschke, Claudia Schirp, Thomas Vietor

**Affiliations:** 1 TU Braunschweig, Institute for Engineering Design, 38106 Braunschweig, Germany; j.busse@tu-braunschweig.de (J.B.); h.watschke@tu-braunschweig.de (H.W.); t.vietor@tu-braunschweig.de (T.V.); 2 Fraunhofer Institute for Wood Research, Wilhelm-Kauditz-Institut WKI, 38108 Braunschweig, Germany; arne.schirp@wki.fraunhofer.de (A.S.); claudia.schirp@wki.fraunhofer.de (C.S.)

**Keywords:** 3D printing, additive manufacturing, material extrusion, continuous fiber-reinforced polymer additive manufacturing, carbon fiber, flax fiber, polylactic acid, design for additive manufacturing

## Abstract

Additive manufacturing, especially material extrusion (MEX), has received a lot of attention recently. The reasons for this are the numerous advantages compared to conventional manufacturing processes, which result in various new possibilities for product development and -design. By applying material layer by layer, parts with complex, load-path optimized geometries can be manufactured at neutral costs. To expand the application fields of MEX, high-strength and simultaneously lightweight materials are required which fulfill the requirements of highly resilient technical parts. For instance, the embedding of continuous carbon and flax fibers in a polymer matrix offers great potential for this. To achieve the highest possible variability with regard to the material combinations while ensuring simple and economical production, the fiber–matrix bonding should be carried out in one process step together with the actual parts manufacture. This paper deals with the adaptation and improvement of the 3D printer on the one hand and the characterization of 3D printed test specimens based on carbon and flax fibers on the other hand. For this purpose, the print head development for in-situ processing of contin uous fiber-reinforced parts with improved mechanical properties is described. It was determined that compared to neat polylactic acid (PLA), the continuous fiber-reinforced test specimens achieve up to 430% higher tensile strength and 890% higher tensile modulus for the carbon fiber reinforcement and an increase of up to 325% in tensile strength and 570% in tensile modulus for the flax fibers. Similar improvements in performance were achieved in the bending tests.

## 1. Introduction

Additive manufacturing (AM) processes, especially material extrusion (MEX), have achieved major technological advances in recent years. Only a few years ago, MEX was used almost exclusively to manufacture prototypes, but this has changed due to the further development of the process and the increasing variety of technical materials so that functional parts and end-use products are also increasingly being manufactured by AM.

Additive manufacturing processes are characterized by a layer-by-layer build-up process, which makes it possible to realize new types of design freedom in part development [1]. Due to this layer-wise process and the associated anisotropy of the parts, additively manufactured parts sometimes only inadequately meet the requirements for mechanical load-bearing capacity. As a result, initial investigations and experiments have been carried out in the past on embedding continuous fibers in additively manufactured parts, in order to achieve an increase in mechanical load-bearing capacity. In particular, material extrusion offers great potential for this, because on the one hand, a continuous fiber–polymer compound can be processed as a filament (prepreg), and on the other hand, a fiber could also be embedded in the polymer matrix directly in the printing process (in-situ), thus creating a fiber-reinforced part in both cases without significant additional effort. In this way, the mechanical load-bearing capacity has already been increased by several hundred percent in both tensile and flexural tests [2,3,4,5,6,7].

However, processing continuous fibers using additive manufacturing techniques also poses challenges to the process of ensuring a robust and consistent 3D printing process. Especially, the in-situ processing of continuous fibers by material extrusion is not easily possible, as the embedding of the continuous fiber into the filament strand only becomes feasible with adaptations of conventional MEX printers. Although there are already concepts from several companies such as Markforged (Watertown, MA, USA), Anisoprint (Moscow, Russia), or Shaanxi Fibertech Technology (Shaanxi, China), these equipment manufacturers do not use in-situ processes but dual extrusion systems in which nylon is first extruded and then a carbon fiber roving, also coated with nylon, is applied to the nylon layer [8,9,10,11]. The in-situ processing of continuous fibers, however, has some advantages over these methods, such as higher flexibility with regard to different material combinations (fiber and polymer). For this reason, a few researchers have already attempted to process continuous fiber materials in-situ using MEX [2,3,5,6,8,12,13,14,15,16,17,18].

### 1.1. Fundamentals of Fiber-Reinforced Additive Manufacturing

In the field of additive manufacturing of continuous fiber-reinforced parts, several approaches have been published recently [2,3,5,6,8,12,13,14,15,16,17,18]. Also, in the field of fiber embedding in epoxy resin, many insights have been gained in recent years that can be applied to additive manufacturing of continuous fibers [19,20,21,22,23,24,25,26,27].

Composite production before the printing process is the simplest method for producing fiber-reinforced parts. In this approach, a pre-impregnated fiber matrix filament (prepreg) is fed to the print head and plasticized in the hot end during the printing process. The plasticization of the matrix polymer in the hot end makes the fiber–matrix filament deformable, and it can be applied in a defined manner to the build platform through the nozzle of the print head. The major advantage of this process is the simple material supply in the form of a continuous fiber filament, which enables processing on conventional MEX equipment. The disadvantage is the high price of the currently available continuous fiber composite filaments, which limits their use to only a few applications due to cost-effectiveness. In addition, the fiber and matrix materials are subjected to additional thermal stress during prepreg production, which has a negative effect on natural fiber materials in particular. This process principle is discussed by several researchers such as Zhang et al. [28], Hu et al. [29] and Vaneker [30].

Alternatively, the bonding between continuous fiber and polymer matrix can be generated within the print head. This process principle is often referred to as in-situ composite production. The in-situ production of continuous fiber-reinforced parts does require an adaptation of the MEX system. However, this method offers significantly higher potential. This process has already been investigated by several researchers such as Fischer et al. [31], Peng et al. [32], Matsuzaki et al. [3], Tian et al. [4,5], Yin et al. [33] and Prüß and Vietor [34].

Matsuziku et al. [3] have developed a print head for in-situ processing of carbon and jute continuous fibers, which has a filament strand feed from the top and a fiber feed from the side. Compounding of the fiber–matrix composite occurs just before extrusion through the nozzle. In this way, Matsuziku et al. were able to produce carbon fiber-reinforced specimens with a fiber volume fraction of 6.6%, resulting in tensile strength of 185.2 MPa. The jute fibers were able to achieve a tensile strength of 57.1 MPa with a fiber volume fraction of 6.1%.

LeDuigou et al. [7] processed a continuous flax fiber filament by MEX process and were able to produce test specimens with a height of 1 mm. The fiber volume fraction of the flax fiber specimens is 30.4%, resulting in a tensile strength of 253.7 MPa.

Akhoundi et al. [17] have summarized a review on the results of continuous fiber-reinforced parts from different researchers. This overview shows that some fiber materials could already be processed in-situ using MEX processes, but the fibers also have mechanical peculiarities; therefore, a universally applicable print head has not yet been developed. Interestingly, most of the tests were carried out using continuous carbon or continuous glass fiber materials. Natural fibers such as jute or flax have so far only been processed and investigated in rudimentary form.

Prüß and Vietor [34] have analyzed different additive manufacturing processes and investigated their suitability for embedding fiber reinforcements. The result is that only MEX and laminated object manufacturing (LOM) are suitable for embedding continuous fibers. In addition, Prüß et al. provide design guidelines that should take into account the potentials and restrictions of continuous fiber 3D printing during part design.

In summary, in the field of processing continuous fibers using MEX technology, some experiments and investigations have already taken place and it has already been shown that this process is suitable for embedding continuous fibers. However, the processing of natural fibers has not been given as much attention so far, which is not surprising due to their lower strengths. Thus, if recycling aspects and other environmental influences are taken into account, the use of natural fibers has a high potential for producing fully compostable continuous fiber-reinforced plastic parts.

### 1.2. Aims and Scope

There are several experimental possibilities for processing continuous fibers by means of additive manufacturing processes, as already mentioned. Besides, a significant increase in the mechanical load capacity has been successfully demonstrated by (continuous) fiber-reinforcment. Nevertheless, there is considerable potential for improvement in this research field in terms of material diversity and flexibility in material selection. In addition, the use of natural fibers has so far only been rudimentarily investigated in the context of continuous fiber-reinforced AM. Adapted MEX printing systems capable of processing carbon or glass continuous fibers already exist, but these production machines cannot easily be adapted for universal material use.

For this reason, a modified MEX 3D printer will be presented, which, in addition to processing continuous carbon fibers, is also capable of processing natural fiber materials without conversion or other efforts. Furthermore, the printhead is designed in such a way that other fibers, such as glass fibers, can also be processed. With the help of this manufacturing system, specimens with a height of 4 mm are produced which are then subjected to tensile and flexural tests. In order to make the range of specimens as extensive as possible, both prepreg (flax and carbon) specimens and composites in which continuous flax or carbon fibers are combined with the matrix (PLA) in-situ are investigated in this paper. The flax fiber specimens are additionally varied in the fiber volume fraction in order to be able to investigate the influence of the fiber volume on composite properties. Finally, the tensile and flexural test results are discussed.

## 2. Materials and Methods

### 2.1. Machine and Process for Continuous Fiber-Reinforced Additive Manufacturing

**Figure 1:**
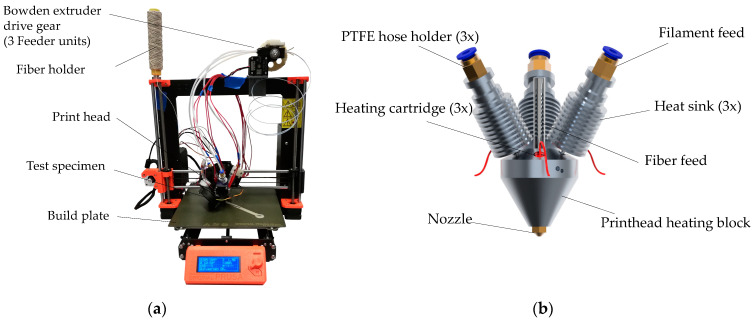
Adaptions and improvements of the 3D printer: (**a**) Overview of the 3D printer modified for the processing of continuous fiber-reinforced polymers; (**b**) Rendering of the developed print head.

The process-integrated (in-situ) processing of continuous fiber-reinforced polymers by means of additive manufacturing, especially material extrusion, places special demands on the manufacturing machine. For this reason, a print head for in-situ fiber-reinforced AM for the existing Prusa I3 MK3S 3D printer (Prague, Czech Republic) was developed—see Figure 1. This print head can process a wide range of polymers and is capable of fusing the polymer matrix with a variety of fibers directly in the print head. In the context of this work, only polylactic acid (PLA) is used as the polymer matrix. Flax and carbon fibers are used as reinforcement materials in this work. These are either embedded in a polymer matrix before printing (prepreg filament) or are embedded in the polymer matrix during production within the print head (in-situ filament). Prepreg filaments were produced using a continuous extrusion process. The modified printer for processing continuous fibers is shown in Figure 1a.

#### 2.1.1. Print Head Development for Processing Continuous Fiber-Reinforced Polymers

In order to implement the print head development for processing continuous fiber-reinforced polymers efficiently and in a goal-oriented manner, the requirements for the print head were analyzed in a first step. The main task of the multi-material print head is to produce and process a fiber-reinforced (carbon and flax) continuous extrusion strand. Since the fibers are relatively limp and flexible compared to the matrix polymer filament, the carbon and flax fiber rovings cannot be pushed directly into the melting chamber of the print head. A promising approach is to transport the fiber through the melt flow of the matrix polymer, which has been tested in other laboratory environments such as [3,17,34]. For a homogeneous compounding process, in this case, three synchronously driven filament strands are directed into the print head at an angle of 30°. The fiber roving is fed in centrally. The filament feeds were used with E3D V6 cold ends and heat sinks to ensure that the filament melting process only occurs within the melting zone. An M8 screw with a stepped hole was used to hold the fiber roving. To minimize mold leakage and friction, a seal made of polytetrafluoroethylene (PTFE) tubing with an inner diameter of 0.5 mm was inserted into the hole inside the screw, see Figure 1b.

To provide the heating energy needed to plasticize the three filament strands, three heating cartridges are used for uniform heating. For this reason, an additional power supply unit is used. A standard nozzle geometry is utilized with an inner diameter of 1.0 mm. In order not to damage the fiber rovings, the inner edge of the nozzle was rounded by grinding.

#### 2.1.2. Development of the Filament Feed and the Cooling System

Figure 1b shows that the filament feed into the print head is symmetrical with three filament strands. As already described, the filament must be fed synchronously into the print head to ensure a constant printing process. The existing 3D printer from Prusa3D (Prague, Czech Republic) has a direct extruder whose material feed is not suitable for simultaneous feeding of multiple filament strands. For this reason, the direct extrusion system would need to be adapted, but this would result in additional feeder stepper motors. This measure would significantly increase the control complexity and the weight of the print head. To keep the weight of the print head as low as possible and thus the agility of the printing system as high as possible, a suitable feeder system based on a Bowden extruder was developed. The Bowden extruder can feed the three filament strands into the print head by Polytetrafluoroethylene (PTFE) Bowden tubes with only one drive shaft and one extruder motor. The entire feeder system can be mounted on the frame of the printer, which means that there is no additional weight on the print head.

In addition to the filament feeder, the cooling system of the print head unit is also crucial to ensure an uninterrupted printing process. Cooling is required for each heat sink on the filament feed to prevent the filament from melting too early and becoming soft. This would result in no targeted material feed into the print head. Furthermore, the extruded composite material must also be cooled, which could be achieved with the help of the existing fan and a modified airflow. A powerful server fan with a 3D printed air outlet, which ensures that all three heat sinks are sufficiently cooled, cools the filament feed. To mount the print head unit to the 3D printer, an adapter was designed to connect the Prusa’s x-axis carriage to the top of the two rear cold ends for an optimal fit. A new mount for the printer’s IR leveling sensor was also designed and installed to ensure a repeatable initial bed leveling.

### 2.2. Additive Manufacturing of Continuous Fiber-Reinforced Test Specimens

#### 2.2.1. Specimen Design

For the additive manufacturing of continuous fiber-reinforced polymer parts, a test specimen was developed, which fulfils the process-specific design requirements limitations, e.g., minimum radii, overhangs and continuous extrusion paths. The difficulty is in the path planning, as the part must be manufacturable without lifting the print head. For the development of AM parts, a systematic development using various design tools is important to support Ideation and ensure a purposeful design [35,36,37]. Nevertheless, AM potentials must be considered in the early stages of product development; otherwise, the advantages of AM such as design freedom cannot be fully exploited [38]. For this reason, the potential and limitations of additive manufacturing with respect to continuous fiber fabrication and processing were considered in the development of the specimen, to ensure additive processability using material extrusion and the developed print head.

In order to be able to manufacture a specimen suitable for the planned tensile and flexural tests, several specimen types were tested. The initial shape of the specimens is based on specimen type 1A according to DIN EN ISO 3167—see also [39]. Specimen 1A is divided into a central section and two clamping sections, with the central section having a width of 10 mm, a length of 60 mm and an overall length of 150 mm. These dimensions correspond to DIN EN ISO 527 for tensile tests—see also [40]. The clamping sections, on the other hand, are not suitable for the additive manufacturing of continuous fibers, since the radii of the area are too small to allow these areas to be continuously manufactured fiber reinforcement. For this reason, the clamping sections were first modified and, finally, the newly developed specimen replaced the previously used specimen.

**Figure 2:**
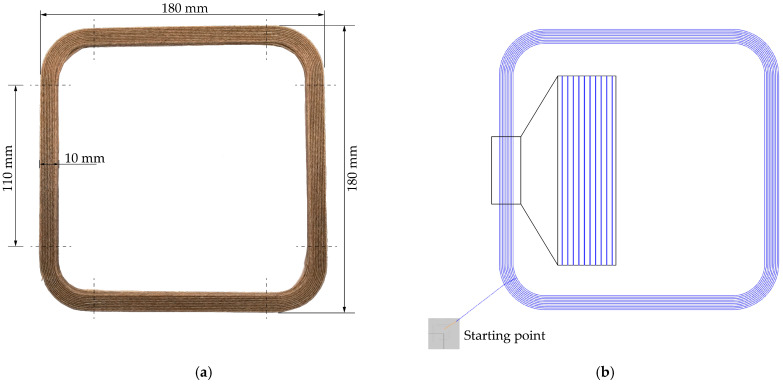
(**a**) Newly developed specimen for material characterization of continuous fiber-reinforced parts; (**b**) Graphical representation of the optimized path planning of the square profile with rounded corners.

Figure 2 shows the newly developed specimen with embedded flax fibers. The rectangular shape with rounded corners enables a more robust, continuous fiber-reinforced additive manufacturing of the specimen. The unique feature of this type of test specimen is that four test specimens can be used directly for tensile and flexural tests, since only the straight edges can be used for material characterization. The test specimens have a width of 10 mm and a length of 110 mm and thus comply with DIN EN ISO 527 [40]. The four test specimens are obtained by cutting them from the rectangle—see dashed lines. The corners were used to determine the fiber volume fraction.

#### 2.2.2. Additive Manufacturing Process and Path Planning

The continuous fiber-reinforced parts are produced by material extrusion using the adapted 3D printer. Material extrusion offers good possibilities for in-situ processing of continuous fibers, since the melting of the filament strand allows direct embedding of the fiber in the polymer matrix. In addition, material extrusion uses nozzles for defined extrusion onto the build platform, through which the fiber–polymer compound can be selectively processed. However, the production of continuous fiber-reinforced parts also places special demands on the 3D printing process, as an uninterrupted application of the fiber must be ensured, which means additional complexity in the slicing process. Furthermore, in-situ continuous printing of the fiber in combination with the realization of AM potentials is at the limit of what is practically possible.

For this reason, the process parameters in the printing process must be precisely adapted to the desired component. Otherwise, no successful production of continuous fiber parts is possible. As soon as the environmental conditions (ambient temperature, humidity) or the process parameters (filament flow, cooling, print head temperature) are not correctly matched to the application, manufacturing errors occur, such as fiber breakage or incorrect application to the build platform. The leveling of the printer, which means the distance between the nozzle and the build platform, is also particularly important for a successful printing process. If the distance between the nozzle and the build platform is too large, the contact pressure is too low and the fiber is pulled off again. If printing begins with too hard leveling, a few centimeters of the fiber–polymer compound can be deposited before the friction between the nozzle edge and the build platform severs the fiber. The process parameters used are shown in Table 1.

The flax fibers (100% Flax long fiber wet spun roving (nature) 200 tex, Franz Holstein GmbH (Tönisvorst, Germany)) and carbon fibers (Carbon fiber NF-3 Roving, Carbon-Werke Weißgerber GmbH & Co. KG (Wallerstein, Germany)) are embedded in a polylactic acid (PLA)-based polymer matrix (Ingeo Biopolymer 3D870, NatureWorks (Minnetonka, Minnesota, USA)) and were used to produce the continuous fiber compound, which is then applied to the build platform through the nozzle on the print head. The resulting part shape depends not only on the part design but also on the slicing process, since the deposition of the fiber in the part must be determined in the slicing process.

In total, 32 test specimens with flax fibers, 24 test specimens with carbon fibers and 8 PLA specimens as reference samples were manufactured, with resulting build times between 80 and 270 min per test specimen. The specimens were sliced with Simplify3D^®^ (4.1.2, Simplify3D, LLC, Cincinnati, OH, USA, 2020) and manufactured on the modified Prusa I3 MK3S (Prague, Czech Republic). In addition, in some cases, the number of fiber layers was varied while keeping the specimen height constant, to determine if higher strengths could be achieved when the fiber volume fraction of the specimens was increased. The test specimens were also made from PLA only, without embedded fibers, in order to obtain reference specimens as well. Prepregs made with flax and carbon fibers were prepared by embedding the fibers in a PLA matrix using a single-screw extruder. Table 2 shows the different types of composites prepared and the number of all test specimens.

Table 2 also shows that prepreg filaments with continuous flax and carbon fibers were manufactured for comparison purposes by using an unmodified Prusa I3 MK3S and the process parameters shown in Table 1. It should also be mentioned that the in-situ Flax specimen consists of 5 layers of fibers at a specimen height of 4 mm. The in-situ Flax 6 and 8 layer specimens contain 6 and 8 layers of fibers, respectively, with the same specimen height. The layer height of the individual specimen types varies between 0.5, 0.66 and 0.8 mm to ensure the same specimen height when embedding a higher number of fiber layers. This can also be seen in the fiber volume fractions in Table 2. These specimens are also subjected to tensile and flexural tests so that a comparison with the in-situ specimens is possible, see Section 3.

It is important that a continuous line, which makes printing without lifting the print head possible, can describe the layer of the part. To achieve this, NC-viewer is used to adapt the resulting path planning again. Figure 2b shows the path planning of the newly developed test specimen. It can be seen that there are no overlapping lines, making the part manufacturable with embedded continuous fiber. In addition, the raster angle orientation of the specimens can be seen. The specimen is continuously built up with an application of the fiber–matrix strand from the outside to the inside. During a layer change, the application changes from the inside to the outside, due to printing without lifting the print head. In this way, specimens are manufactured with a raster angle orientation of 0°.

### 2.3. Mechanical, Optical and Thermal Material Characterization

Following the additive manufacturing of test specimens with different fiber-reinforced polymers, the mechanical properties of these specimens have to be tested. For this purpose, the test specimens are subjected to tensile and flexural tests to determine the tensile and flexural strengths and modulii of all test specimens. The use of reinforcing fibers promises a significant increase in strength and bending stiffness compared with conventional plastics. This improvement in mechanical part properties is particularly evident in parts subjected to tensile or shear loads parallel to the fiber direction. Accordingly, reinforcing fibers such as carbon fiber or aramid are preferably used in corresponding parts where the main fiber directions of the uni- or multidirectional composite coincide with the main load paths.

To compare the tensile and flexural properties of the manufactured composite parts with those of other fiber-reinforced parts, tensile tests and flexural tests are carried out using DIN EN ISO 527-4, 178 and 14125 [40,41,42], respectively. The preferred test specimen according to DIN EN ISO 178 [42] has a length of 80 mm, a width of 10 mm and a height of 4 mm. It thus corresponds exactly to the dimensions of the narrow center section of the universal specimen according to DIN EN ISO 3167 [39,40,41,42].

DIN EN ISO 527-4 [41] (Determination of tensile properties—Part 5: Test conditions for unidirectional fiber-reinforced plastic composites) refers to the use of a selection of two specimen types. Both are to be cut from sheet materials, have an overall length of 250 mm, a distance between the force application elements of 115 mm and a gauge length of 50 mm. They differ in their width and the orientation of the unidirectional fibers they contain. If the reinforcing fibers are arranged in the longitudinal direction, specimen type A is provided with a specimen width of 15 mm and a thickness of 1 mm. If the fibers run transversely to the tensile direction, specimen type B with twice the thickness of 2 mm and a width of 25 mm should be used. The force application elements at the restraining ends of the specimens are cut from a glass fiber epoxy sheet material with a thickness between 0.5 and 2 mm, and bonded to the specimen with a highly elastic adhesive so that the orientation of the fibers of the glass fiber stickers is aligned at 45° to the tensile direction. [40] For an exact determination of the tensile modulus between 0.05% and 0.25% elongation, a test speed of 1 mm/min is applied at the beginning of the tensile test. At 0.5% elongation the test speed is doubled to 2 mm/min according to DIN EN ISO 527-4 [40].

**Figure 3:**
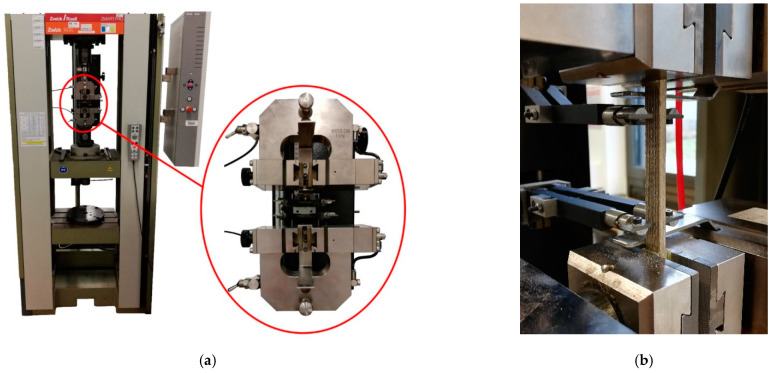
Material characterization: (**a**) Zwick/Roell 1474 universal testing machine; (**b**) Close-up of an in-situ flax fiber specimen during tensile testing.

In the further course of the work, the Zwick/Roell 1474 universal testing machine (Ulm, Germany) shown in Figure 3 is used for all mechanical tests. The figure also shows separately the measuring and clamping device used in the tensile test according to DIN EN ISO 527-4 [40]. It consists of the upper and lower clamping jaws and a displacement transducer for recording specimen strain during the tensile test. The flexural tests according to DIN EN ISO 178 [42] or 14,125 [41] are carried out at a test speed of 2 mm/s.

Thermal decomposition of PLA, carbon fibers and 3D-printed PLA–carbon fiber composites was investigated by means of thermogravimetric analysis according to DIN EN ISO 11358-1 [43] (TGA) using a TGA/DSC 1 (Mettler-Toledo AG, Schwerzenbach, Switzerland). Samples (approx. 10 mg) were weighed into a TGA crucible and subsequently placed in the TGA instrument. Samples were heated from 25 °C to 1000 °C at a heating rate of 10 K·min^−1^ under a constant airflow of 50 mL·min^−1^.

The aim of the test is to calculate the amount of carbon fibers in the PLA–carbon fiber composites based on complete thermal degradation of the matrix polymer PLA. This is possible because the thermal decomposition steps of PLA and carbon fibers do not overlap over a wide temperature range. Due to the low decomposition temperature of flax fibers, this method can only be used for carbon fiber-reinforced samples. To determine the volume fraction of the flax fibers in the PLA–flax fiber composites, a 3D surface profilometer type VR5300 from Keyence (Neu-Isenburg, Germany) was used. For this purpose, microscope images were evaluated and the fiber volume fraction was determined by calculation using the cross-section of the fibers and the sample.

## 3. Results and Discussion

### 3.1. Material Characterization of the Continuous Fiber-Reinforced Composites Processed by Additive Manufacturing

The material characterization was carried out as described in Section 2.3. with the in-situ flax fiber specimens, the prepreg flax fiber specimens and with the in-situ carbon fiber specimens and carbon specimens (prepreg). For this purpose, the flexural tests are explained first, so that the maximum flexural strength of the individual specimens can be measured. This is followed by the tensile test of the specimens. Finally, the fiber volume fractions of the specimens are determined with the aid of thermogravimetric analysis and 3D surface profilometer technique.

#### 3.1.1. Flexural Tests

The flexural tests within the contribution were carried out according to DIN EN ISO 178—see also [42]. The in-situ flax, in-situ carbon specimens and the prepreg specimens were tested to determine the flexural strength and flexural moduli. For comparison purposes, samples made of PLA without embedded fibers were also tested.

Table 3 shows the flexural tests of the different specimens. The mean values of the individual specimens in terms of maximum elongation, flexural strength and flexural modulus including their standard deviations are shown.

**Figure 4:**
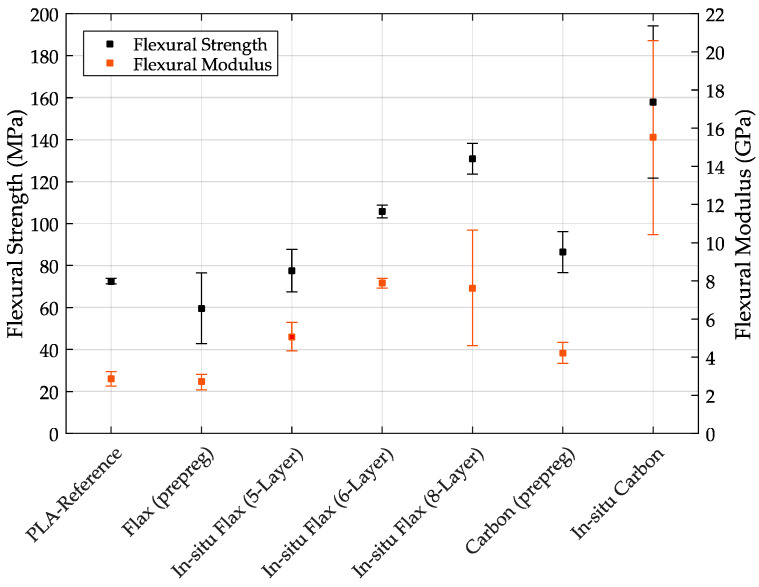
Flexural strength and modulus with standard deviation for all test specimen types.

Figure 4 shows the respective specimens on the x-axis and the flexural strength (left) and flexural modulus (right) on the y-axes. It can be seen that the reference specimen made of PLA and the flax fiber (prepreg) specimen has the lowest flexural strengths. It is particularly surprising that the flax fiber (prepreg) sample, with an average bending strength of 59.64 MPa, actually performs significantly worse compared to the PLA specimen (without fiber). The in-situ flax fiber samples, on the other hand, have higher flexural strengths than the specimens made of PLA due to the embedding of the flax fiber. This behavior was also expected for the flax (prepreg) specimens. The in-situ flax fiber sample with 8-layers of fibers, at a constant sample height, exhibits particularly high flexural strengths with a value of 130.99 MPa. However, the in-situ flax fiber sample with 6-layers also achieves a value of 105.75 MPa.

These significant differences are due to the higher fiber volume fraction in the 8-layer composite samples, and indicate that the fibers are well integrated into the polymer matrix. Otherwise, delamination of the specimens could be expected.

The carbon fiber specimens also achieve higher flexural strengths than the PLA reference specimen do, although again the in-situ specimens reach significantly higher flexural strengths than the carbon specimens (prepreg). The carbon fiber specimens (prepreg) have slightly higher values than the in-situ flax fiber 5-Layer specimens with 86.4 MPa. The in-situ carbon fiber specimen achieved the highest flexural strengths in this study with a value of 157.9 MPa. However, it should be mentioned here that the standard deviation of the in-situ carbon specimens is very large, which is probably due to difficult fiber–matrix bonding, since the carbon fibers have a coating that makes fiber–matrix bonding difficult.

In summary, it was determined that the embedding of flax and carbon fibers can have a significant positive effect on the flexural strengths and the flexural moduli. It is found that especially the in-situ variants achieved significantly higher values, although the carbon specimens (prepreg) could also achieve higher values than the PLA reference specimens as could be expected due to fiber inclusion. In general, the prepreg specimens performed significantly worse than the in-situ variants, which is probably due to poor fiber embedding in the polymer matrix. Whether this insufficient embedding already occurred in the precoated filament used, or in the printing process, is difficult to say. However, this behavior is probably due to the prepreg production (insufficient amount of polymer matrix for coating the fiber, no uniform embedding of the fiber).

**Figure 5:**
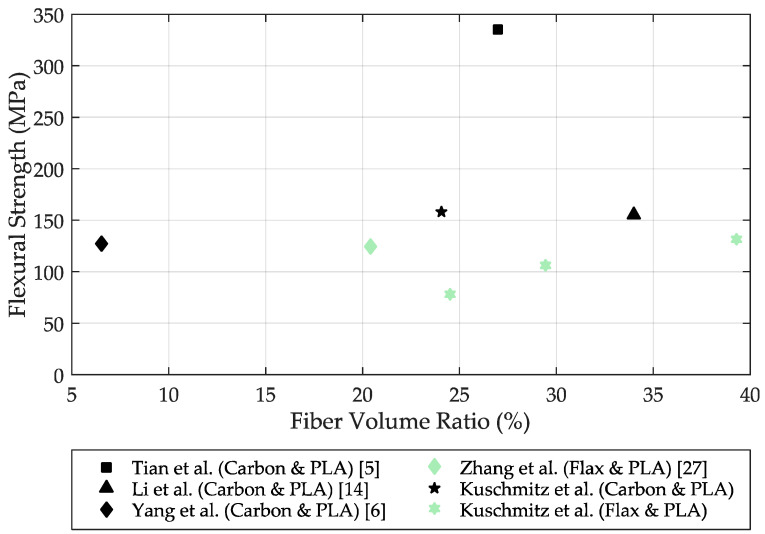
Comparison of the flexural strength of in-situ carbon and in-situ flax fiber-reinforced specimens. The black symbols indicate the carbon fiber specimens, the green symbols indicate the natural fiber (flax) specimens.

Figure 5 shows a comparison of some in-situ printed continuous fiber-reinforced test specimens. The black symbols indicate the carbon fiber specimens and the green symbols indicate the natural fiber specimens (flax). The figure shows the flexural strength as a function of the fiber volume fraction of each specimen. It can be seen, for example, that Tian et al. [5] were able to produce a carbon fiber-reinforced specimen with a fiber volume fraction of 27% and a resulting flexural strength of 335 MPa. Li et al. [14] were able to produce a carbon fiber specimen with a fiber volume fraction of 34% and a flexural strength of 156 MPa. Compared with the highest flexural strength of the carbon samples in this paper, the values of flexural strength are relatively close, but the fiber volume content is about 10% lower compared to Li et al. with a value of 24.04%. This difference in fiber volume content may be due to the specimen height, which is 4 mm for Kuschmitz et al. and 2 mm for Li et al. [14]

Flexural strengths for in-situ fabricated natural fiber-reinforced specimens have been scarcely reported in the literature. Zhang et al. [27] have embedded in-situ continuous flax fibers in a PLA matrix. The fiber volume fraction of the specimen is about 20.4%, achieving a flexural strength of 125 MPa. The continuous flax fiber samples by Kuschmitz et al. in this paper were able to achieve a flexural strength of 77.72 MPa with a fiber volume content of 24.54%. With a fiber volume content of 39.52%, even a flexural strength of 130.99 MPa could be obtained. Thus, Zhang et al. were able to achieve a similar flexural strength at a lower fiber volume fraction. This could be due to poorer fiber–matrix bonding in our printing trials; for example, due to the coating of the carbon fibers. However, it is more likely that variation in specimen height is responsible for the observed differences. In the current research project, printed test specimens were significantly higher compared to Zhang et al.; hence, there is greater potential for defects during the printing process of the individual layers. In general, the bonding between the individual layers also has a decisive influence on the flexural strength. For further improvement of strength properties, coupling agents could potentially be included. Another parameter which influences flexural strength is the fiber alignment. The better the alignment, the higher the resulting strength.

#### 3.1.2. Tensile Tests

**Figure 6:**
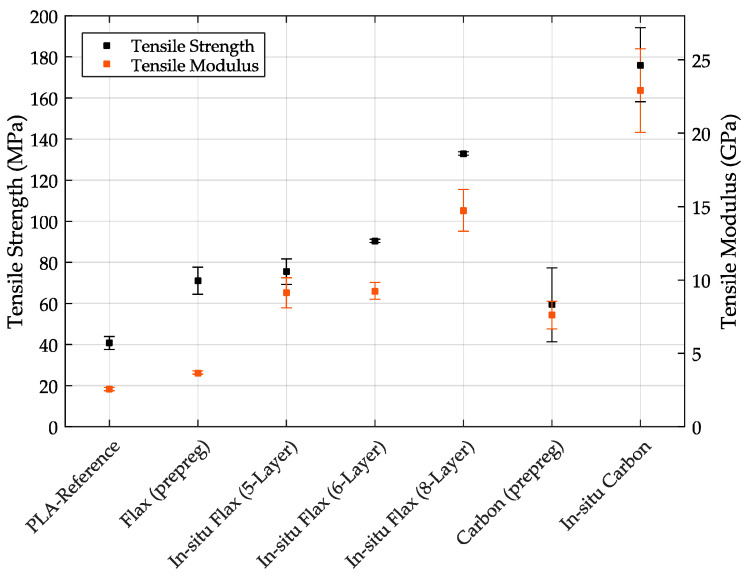
Tensile strength and modulus with standard deviation for all test specimen types.

The tensile tests were also performed with the seven different specimen types. Table 4 shows the measured mean values of the respective specimen for the maximum force, tensile strength and tensile modulus. In addition, the mean values of the specimens are also plotted in Figure 6, including the standard deviations.

It can be seen that also in the tensile tests, the reference specimens made of PLA with 40.75 MPa show significantly lower tensile strengths compared to the fiber-reinforced specimens. The flax fiber specimens (prepreg and in-situ (5-Layer)) show similar results, with the in-situ specimen achieving approximately 4.5 MPa higher tensile strengths (75.47 MPa) compared to the prepreg composites. Similar to the flexural tests, the in-situ flax fiber specimens with 6 and 8 layers of flax fibers were able to achieve significantly higher tensile strengths, with values of 90.47 MPa (6-layer) and 132.9 MPa (8-layer) compared to the prepregs and the in-situ flax fiber with 5 layers. The standard deviations of the 6 and 8 layer in-situ flax fiber specimens are also very low. Similarly good behavior was observed for the tensile moduli. The lowest tensile modulus in the sample range is achieved by the reference sample made of PLA with a value of 2.57 GPa. However, the flax (prepreg) specimen shows a slightly higher value of 3.69 GPa. Interestingly, both the in-situ flax sample (5-layer) and the in-situ flax (6-layer) sample achieved nearly identical tensile moduli of 9.12 and 9.26 GPa, respectively. It appears that the second highest tensile moduli were reached with 6 layers of flax fibers under the conditions of the investigations. The highest tensile modulus of the natural fiber specimens was achieved with the in-situ flax specimen (8-layer) at 14.75 GPa.

The carbon fiber specimens showed significantly different tensile strengths. The carbon (prepreg) specimens have the lowest tensile strength, following behind the reference specimen (PLA) with a value of 59.35 MPa, whereas the in-situ carbon sample could achieve the highest tensile strength with a value of 176.2 MPa. Similar results have been measured for the tensile moduli. The third lowest tensile MOE overall was achieved for the carbon specimen (prepreg) with 7.61 GPa. The in-situ carbon sample, on the other hand, was able to achieve the highest tensile modulus with a value of 22.9 GPa.

Overall, it was also shown in the tensile tests that the embedding of flax and carbon fibers resulted in a positive effect in terms of tensile strength and tensile modulus. Especially, it was found that the in-situ variants could achieve better results than the prepreg specimens. However, the prepreg flax fiber samples achieved significantly higher values than in the flexural tests.

It is noticeable that the standard deviations of the carbon samples are significantly higher than the standard deviations of the flax fiber samples, which is probably due to the coating of the carbon fibers. The coating makes the fiber–matrix bond comparatively more difficult to produce, which is expected to result in poorer fiber bonding in the compound. Furthermore, the determined values could also be higher if the fiber–matrix bond is optimized. The standard deviation of the flax fiber samples is comparatively low; however, few variations occurred in the determination of flexural and tensile strengths and moduli. These fluctuations are due to the difficulty in making the manufacturing process robust and qualitatively constant. At present, variable polymer matrix flow occurs more frequently, which is due to an inhomogeneous coating of the individual fibers.

Nevertheless, it could be shown that the embedding of fibers directly in the printing process is possible and that this variant even achieved better results. In particular, the specimens with the highest possible fiber volume fraction combined with good embedding in the polymer matrix were able to achieve significantly better results than the PLA reference or the prepreg specimens. For example, at least a 30% increase in flexural strength could be achieved in the in-situ flax fiber specimens compared to the flax (prepreg) specimens. With an increase in the fiber volume fraction, the flexural strength can be more than doubled. However, the standard deviations are relatively high in some cases, as a certain scatter could be detected in the measurement. This is due to the difficulty of achieving consistent printing results in the specimens.

**Figure 7:**
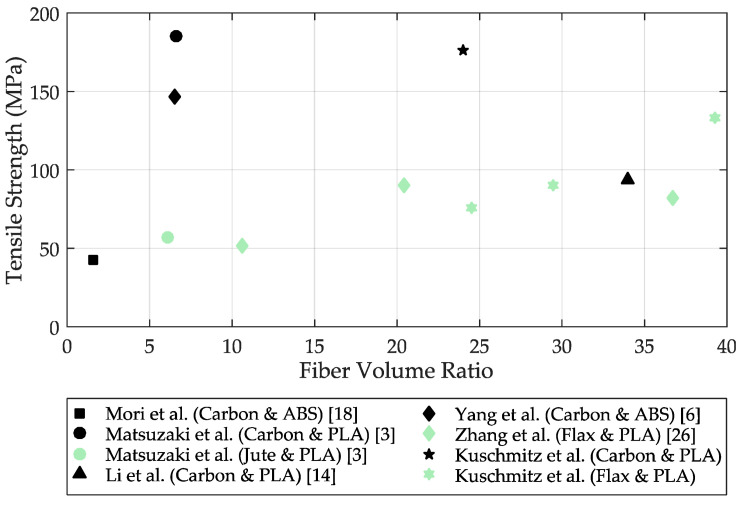
Comparison of the tensile strength of in-situ carbon and in-situ natural fiber-reinforced speci-mens. The black symbols indicate the carbon fiber specimens, the green symbols indicate the natural fiber (jute and flax) specimens.

Figure 7 shows a comparison of some in-situ printed continuous fiber-reinforced test specimens. The black symbols indicate the carbon fiber specimens and the green symbols indicate the natural fiber specimens (jute and flax). The figure shows the tensile strength as a function of the fiber volume fraction of the respective specimen. For example, Mori et al. [18] were able to additively manufacture a carbon fiber-reinforced specimen with a fiber volume fraction of 1.6% and a tensile strength of 43 MPa. Matsuzaki et al. [3] were able to additively fabricate a carbon fiber-reinforced specimen with a fiber volume fraction of 6.6% and a tensile strength of 185.2 MPa. Compared with the highest tensile strength of the carbon specimens in this paper, the values of tensile strength are relatively close; however, the fiber volume fraction in the specimen of Kuschmitz et al. is 24.04%, which is much higher. It can be concluded that Matsuzaki et al. [3] were able to achieve much better fiber–matrix bonding in the specimen; however, these specimens also consist of a much lower fiber fraction at the same height, so good fiber–matrix bonding is much easier to achieve with a much higher polymer matrix fraction.

Zhang et al. [27] also embedded in-situ continuous flax fibers in a PLA matrix and manufactured test specimens with a sample height between 0.8 and 1.2 mm. With a fiber volume fraction of 10.6%, a tensile strength of 51.2 MPa was obtained. By increasing the fiber volume fraction, Zhang et al. were able to achieve a tensile strength of 82 MPa with a fiber volume fraction of 36.7%. The continuous flax fiber specimens by Kuschmitz et al. in this paper were able to achieve a tensile strength of 75.47 MPa with a fiber volume content of 24.54%. With a fiber volume content of 39.27%, even a tensile strength of 132.9 MPa could be achieved.

### 3.2. Analysis of the Investigated Specimens

In order to better understand the results of the tensile and flexural tests, this section first includes a brief inspection of the specimens after the tensile tests, including a short analysis of the fracture site to identify potential fiber matrix defects. In addition, the fiber content in the specimens is determined.

#### 3.2.1. Specimen Inspection after Tensile Testing

**Figure 8:**
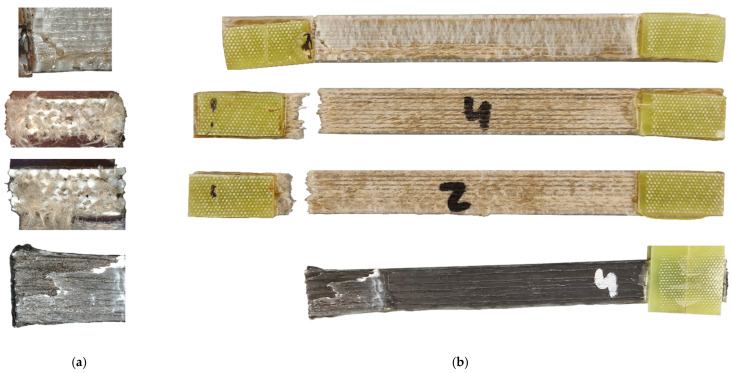
(**b**) Overview of selected in-situ fiber tensile specimens (flax fiber-reinforced specimens 2, 4, 8 and carbon fiber-reinforced specimen 4) and (**a**) the corresponding tested tensile specimens for the investigation of the fracture pattern.

The tensile test has shown that individual specimens sometimes exhibit strong deviations from the average tensile strength of the specimen type. For this reason, it is suspected that this observation is due to poor fiber embedding or poor matrix–fiber bonding as, otherwise, the results should be more consistent. For this reason, the in-situ flax fiber specimens that achieved relatively high standard deviations in the tensile test were examined and illustrated in Figure 8.

In-situ flax fiber specimens 2, 4 and 8 are shown (Figure 8b) as well as in-situ carbon specimen 4 and fracture patterns of the in-situ flax and carbon fiber specimens (Figure 8a). Specimen 2 performed relatively average with a tensile strength of 78.16 MPa. Specimen 4 and 8 represent the worst (specimen 8) and best tensile strength of the sample series with values of 87.28 MPa and 65.50 MPa, respectively. Figure 8 also shows that all specimens are fractured relatively close to the left-hand fixture, which could indicate force transfer from the fixture to the fiber. Additionally, it can be seen that specimens 2 and 4 have a relatively homogeneous fracture pattern with a perpendicular fracture area. There has also been a complete severing of the fibers, indicating a good bond between the fiber and the polymer matrix. Specimen 8, on the other hand, shows a break in the polymer matrix, but the fibers are not damaged. Therefore, it is suspected that the fiber–matrix bonding in sample body 8 is insufficient at the point of breakage and a stair-step-like fracture pattern has occurred in the polymer matrix. During this process, the fibers were likely elongated; however, due to the inadequate bond to the polymer matrix, the fiber did not prevent this fracture pattern. Figure 8a shows the fracture surface of the in-situ flax fiber specimens 4 and 2. It can be seen in both cases that the fibers are well enclosed by the polymer matrix; however, it can also be seen that in some cases the fiber–matrix bond is expandable. This defect explains the different results in the tensile tests. Additionally, it would be desirable if the fiber was more coated with the matrix polymer.

High standard deviations were also observed in the carbon specimens, which again is probably due to insufficient fiber embedding in the polymer matrix. The additional difficulty in processing the carbon fibers is the coating of the fiber, which is an additional obstacle in fiber–matrix bonding. Figure 8b shows in-situ carbon sample 4 which was able to achieve the highest tensile strength in the test series of carbon fibers with a value of 203.46 MPa. However, it can also be seen that the polymer matrix including individual fiber strands was torn out of the fiber–matrix bond. This can be seen especially in Figure 8a below. If it were possible to strengthen this bond, for example by using suitable coupling agents for fiber and matrix, such behavior could possibly be prevented and the tensile strength could probably be significantly increased.

Overall, good tensile strengths were achieved in the tests, which could be significantly increased again by the measures mentioned for the in-situ flax and carbon fiber samples. The flax and carbon (prepreg) specimens would also benefit from this and achieve higher tensile strengths.

#### 3.2.2. Fiber Volume Fractions of the Specimens

Thermogravimetric analysis (TGA) was performed to determine the fiber volume fraction of the specimens. However, this method can only be used for the carbon fiber samples because the flax fibers degrade at a similar temperature range as the polymer matrix, so no change in mass can be meaningfully detected.

**Figure 9:**
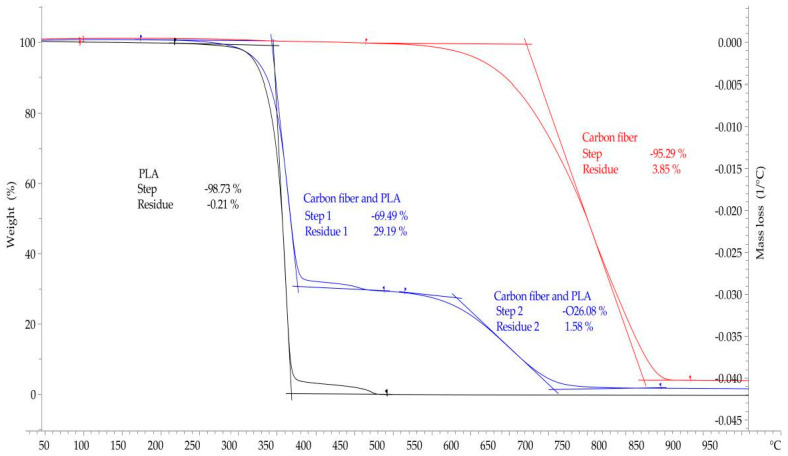
Thermogravimetric analysis of PLA (**black curve**), carbon fiber (**red curve**) and in-situ carbon fiber specimen (blue curve).

Figure 9 shows the TGA of PLA (black curve), of the carbon fiber (red curve) and of the in-situ carbon fiber specimen embedded in PLA (blue curve). It can be seen that at a temperature of about 365 °C, 97% of the mass of the PLA specimen has already been degraded. At a temperature of approx. 470 °C, total decomposition of PLA has occurred. Hence, it can be assumed that in the carbon fiber-PLA curve, residual mass above 470 °C consists of carbon fiber only and does not contain any PLA. The red curve shows the decomposition of the carbon fiber only. It can be seen that at a temperature of 530 °C, the mass fraction of the fiber slowly begins to be reduced. At a temperature of 900 °C, the fiber is completely degraded.

The blue curve indicates the TGA of the in-situ carbon fiber sample. It can be seen that at a temperature of about 370 °C, a significant step is completed and 69.49% of the total weight has been degraded. At this temperature only a minute amount of PLA is left in the specimen. At a temperature of about 470 °C, another small decrease in mass fraction occured.

In summary, the thermogravimetric analysis showed that the carbon fiber samples have a fiber weight fraction of about 26–28% carbon fibers.

To calculate the fiber volume fraction, a fiber volume fraction of 24.04% was determined with a density of 1.8 g/cm³ for the carbon fibers. The calculation was made using the total mass of the specimen, the weight percent of the carbon fibers, and the density of the carbon fiber.

**Figure 10:**
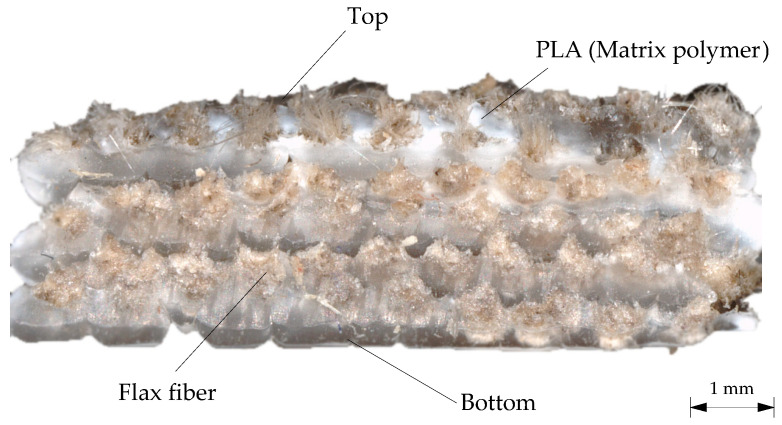
Microscope image of an in-situ flax fiber sample to determine the fiber volume fraction.

The fiber volume fraction of the flax fiber samples was determined both by means of a 3D surface profilometer type VR5300, and analytically since the flax fiber was partly vaporized with the PLA so that a measurement of the fiber content is not possible. For this purpose, microscope images were evaluated on the one hand and the fiber volume fraction was determined computationally via the cross-section of the fibers and the specimen (Figure 10). Compared to the thermogravimetric analysis of the carbon fiber samples, the results obtained are probably less accurate, but they provide a good estimation for comparison.

Analytically, the theoretical fiber volume fractions of the specimens were also determined. The diameter of the flax fiber is 0.5 mm and 10 fiber strands are placed side by side per layer. The specimens are approximately 10 mm wide and 4 mm high. With the aid of these values, the theoretical fiber volume fractions could be determined (Table 2). Since the method using optical measurement technology is susceptible to error, the analytically determined fiber volume fraction was always given in the paper.

## 4. Conclusions and Outlook

In this paper, a print head for in-situ processing of continuous fibers was developed and tested. The print head is capable of printing natural and synthetic continuous fibers with a universal polymer matrix in a conventional MEX process. A total of seven different specimen types were produced, which had flax or carbon fibers embedded in a PLA polymer matrix.

The tensile and flexural tests carried out showed that the in-situ embedding of the continuous fibers resulted in a significant increase in strength. However, it was also found that the fiber–matrix bond could be expanded in places, which would result in even better strength increases. Thus, highly durable functional parts for the automotive industry could be produced with material extrusion in the future. In aircraft manufacturing, it is expected that this additive manufacturing process combined with automated tape laying (ATL) can repair defects in large fiber composite structures [44]. This can significantly reduce repair and maintenance costs in this field.

It has been successfully demonstrated that the processing of continuous fibers by MEX is possible in principle and that the mechanical strength of the parts can be significantly increased in this way. However, it will still take some research before entire parts can be 3D printed with embedded continuous fibers in a meaningful way and be used in automotive engineering or aviation. Besides a robust manufacturing process with an additional cutting mechanism, advanced methods and tools for supporting an automated design process and path planning are necessary.

For this reason, fiber–matrix bonding should be investigated in further studies. It could be shown that poor fiber–matrix bonding leads to severe decimation of tensile and flexural strengths. It is also imperative to fabricate thicker specimens. So far, in many previous investigations, specimens with a maximum height of only 2 mm have been additively manufactured or the fiber fractions of the specimens were low, respectively. Hence, the negative effects of layer apposition can only be scarcely detected, since either too few layers were printed, or achieving sufficient bonding was quite easy due to the low amount of fiber fraction. In addition, the necessary influence of the heated build platform could also be further examined in future research as well as applications to fulfill additive manufacturing of continuous fiber-reinforced parts.

## Figures and Tables

**Table 1:** Process parameters used for the 3D printing of the test specimens for both carbon fiber and flax fiber reinforcement.

Process Parameter		Value
Nozzle diameter	[mm]	1
Nozzle temperature	[°C]	205
Platform adhesion		crepe tape
Platform temperature	[°C]	60
Extrusion speed	[mm/s]	1.75
Print head speed	[mm/s]	1
Layer height	[mm]	0.51 ^1^; 0.66 ^2^; 0.8 ^3^
Extrusion width	[mm]	1

^1^ in-situ Flax (8-Layer) Specimens; ^2^ in-situ Flax (6-Layer) Specimens; ^3^ in-situ Carbon, Carbon (prepreg), in-situ Flax, Flax (prepreg), PLA Specimens.

**Table 2:** Overview of the manufactured test specimens (Figure 2) for flexural and tensile testing. Fiber volume fraction determined using TGA for carbon–PLA composites and optical technique for flax–PLA composites.

Test Specimen	PLA	Flax (Prepreg)	In-Situ Flax (5-Layers)	In-Situ Flax (6 Layers)	In-Situ Flax (8 Layers)	Carbon (Prepreg)	In-Situ Carbon (5-Layers)
Flexural Test	8	4	8	2	2	5	8
Tensile Test	8	4	8	2	2	3	8
Fiber volume fraction ^1^	0 %	9.82%	24.54%	29.45%	39.27%	18.86%	24.04%
Quantity	16	8	16	4	4	8	16

^1^ The fiber volume fraction was determined by thermogravimetric analysis and analytical calculation, Section 3.2.2.

**Table 3:** Average values of the flexural tests for all specimen types.

Test Specimen	Max. Elongation (%)	Flexural Strength (MPa)	Flexural Modulus (GPa)
PLA	3.92 ± 0.24	72.62 ± 1.41	2.86 ± 0.38
Flax (prepreg)	3.83 ± 1.1	59.64 ± 16.82	2.69 ± 0.4
In-Situ Flax 5-Layer	3.82 ± 0.46	77.72 ± 10.13	5.08 ± 0.74
In-Situ Flax 6-layer	3.9 ± 0.22	105.75 ± 3.09	7.88 ± 0.26
In-Situ Flax 8-layer	2.93 ± 0.83	130.99 ± 7.33	7.63 ± 3.04
Carbon (prepreg)	2.74 ± 0.17	86.40 ± 9.68	4.22 ± 0.55
In-Situ Carbon 5-Layer	1.93 ± 0.34	157.9 ± 36.26	15.49 ± 5.1

**Table 4:** Average values of the tensile tests for all specimen types.

Test Specimen	F_max_ (kN)	Tensile Strength (MPa)	Tensile Modulus (GPa)
PLA	1.67 ± 0.14	40.75 ± 3.07	2.57 ± 0.11
Flax (prepreg)	3.54 ± 0.32	71.13 ± 6.60	3.69 ± 0.12
In-Situ Flax 5-Layer	3.60 ± 0.18	75.47 ± 6.19	9.12 ± 1.02
In-Situ Flax 6-layer	4.01 ± 0.13	90.47 ± 0.83	9.26 ± 0.58
In-Situ Flax 8-layer	6.23 ± 0.11	132.90 ± 0.80	14.75 ± 1.42
Carbon (prepreg)	2.90 ± 0.50	59.35 ± 17.67	7.61 ± 0.94
In-Situ Carbon 5-Layer	9.08 ± 0.75	176.20 ± 18.01	22.90 ± 2.85

## Data Availability

The data presented in this study are available on request from the corresponding author.
